# Zonulopathies as Genetic Disorders of the Extracellular Matrix

**DOI:** 10.3390/genes15121632

**Published:** 2024-12-20

**Authors:** Chimwemwe Chipeta, Jose Aragon-Martin, Aman Chandra

**Affiliations:** 1 Department of Ophthalmology, Southend University Hospital, Southend-on-Sea SS0 0RY, UK; c.chipeta@nhs.net; 2 Vision and Eye Research Institute, Anglia Ruskin University, Cambridge CB1 2LZ, UK; 3 Barts & The London School of Medicine and Dentistry, William Harvey Research Institute, Queen Mary University of London, London EC1M 6BQ, UK; j.aragon-martin@imperial.ac.uk

**Keywords:** ectopia lentis, zonulopathy, ciliary zonule, Marfan syndrome

## Abstract

The zonular fibres are formed primarily of fibrillin-1, a large extracellular matrix (ECM) glycoprotein, and also contain other constituents such as LTBP-2, ADAMTSL6, MFAP-2 and EMILIN-1, amongst others. They are critical for sight, holding the crystalline lens in place and being necessary for accommodation. Zonulopathies refer to conditions in which there is a lack or disruption of zonular support to the lens and may clinically be manifested as ectopia lens (EL)—defined as subluxation of the lens outside of the pupillary plane or frank displacement (dislocation) into the vitreous or anterior segment. Genes implicated in EL include those intimately involved in the formation and function of these glycoproteins as well as other genes involved in the extracellular matrix (ECM). As such, genetic pathogenic variants causing EL are primarily disorders of the ECM, causing zonular weakness by (1) directly affecting the protein components of the zonule, (2) affecting proteins involved in the regulation of zonular formation and (3) causing the dysregulation of ECM components leading to progressive zonular weakness. Herein, we discuss the clinical manifestations of zonulopathy and the underlying pathogenetic mechanisms.

## 1. Introduction

The ciliary zonule is a critical structure in the eye. The term zonule is taken from the Latin “zonula”, meaning “little belt”, as it forms a 360-degree belt around the lens anchoring it to the ciliary body. Functionally, it serves to hold the lens in the visual axis and is necessary for the lens to change its shape during accommodation. 

Zonulopathies refer to conditions in which there is a lack or disruption of the zonular support to the lens. When zonular weakness is severe, this may be made manifest clinically by oscillation of the lens (phacodonesis) or ectopia lentis (EL), defined as the subluxation of the lens outside of the pupillary plane or frank displacement (dislocation) into the vitreous or anterior segment. The major structural component of the zonule is fibrillin-1 [1], which is a large extracellular matrix (ECM) glycoprotein, and thus, zonulopathies may be considered primarily ECM disorders affecting fibrillin-1 either directly through pathogenic variants affecting fibrillin-1 or indirectly through defects to proteins intimately involved with fibrillin-1 function in the ECM. Zonulopathies may often be the first presenting feature of a systemic and potentially life-threatening connective tissue disorder such as Marfan syndrome (MFS) [2,3].

Surgically, the zonule is of particular interest due to its importance in cataract surgery. During cataract surgery, the natural lens is removed, with only the outer capsule left intact. This empty capsule is known as the “capsular bag” into which the replacement intraocular lens (IOL) is ideally placed. In patients with zonulopathies, the capsular bag may be unable to maintain the IOL in the visual axis and result in IOL dislocations; the incidence of which is increasing [4,5,6]. 

Herein, we discuss the most pertinent genetic and protein relationships of zonulopathies. The primary structural protein of the ciliary zonule, fibrillin-1, will be described, followed by genetic associations which affect this directly or via other proteins. The phenotypes caused not only affect the ciliary zonule, but also affect the systemic ECM. Through the framework of zonulopathies as ECM disorders, we aim to provide the reader insight into the underlying pathogenic mechanisms of zonulopathies.

## 2. Fibrillin and Structure of the Zonule

**Figure 1:**
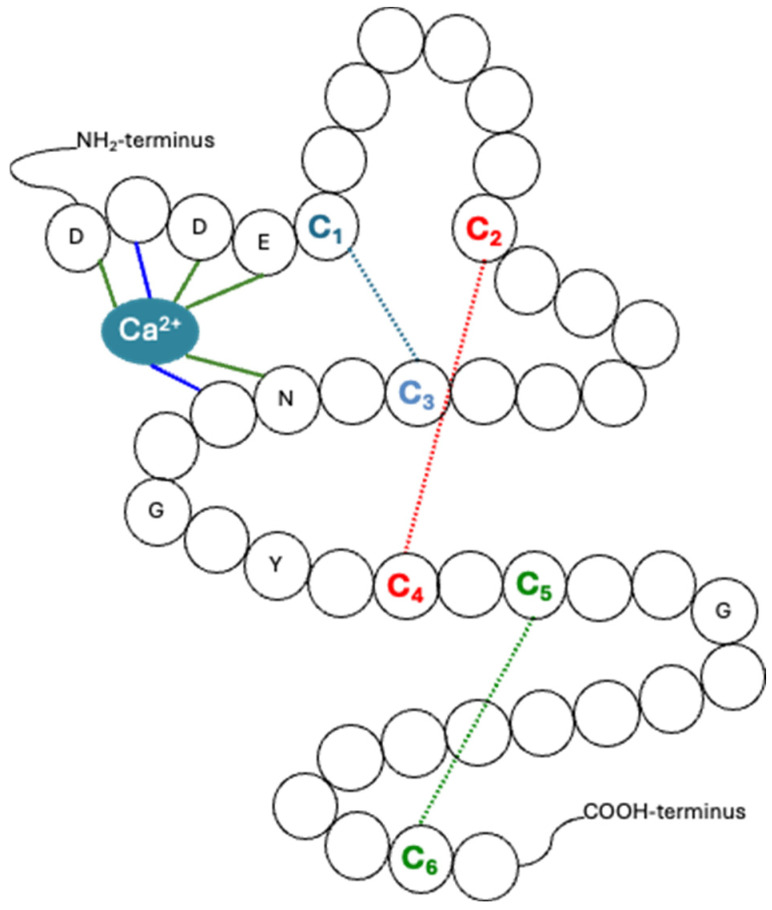
Illustration of a cbEGF-like domain in the fibrillin-1 protein. This is a possible representation of how a fibrillin-1 cbEGF-like domain could look like based on an amino acid sequence and previous published studies [16,17,18]. Calcium (Ca^2+^) is found bound to several amino acids in the calcium-binding region in the cbEGF-like #35 domain, and shows the conserved amino acids for this domain. Disulphide bonds are represented with coloured dotted lines and show the 3 cysteine pairs [C_1_–C_3_ (blue), C_2_–C_4_ (red) and C_5_–C_6_ (green)].

Fibrillins are a major component of the extracellular matrix (ECM). There are three isoforms (fibrillin-1 [OMIM *134797], fibrillin-2 [OMIM *612570], fibrillin-3 [OMIM *608529]) which are composed of repeated epidermal growth factor (EGF) domains, the majority of which are calcium binding (cb-EGF) [7]: fibrillin-1 [4× EGF-like and 43× cbEGF-like] [8], fibrillin-2 [4× EGF-like and 43× cbEGF-like] [9], and fibrillin-3 [4× EGF-like and 40× cbEGF-like] [10]). Each of these have cysteine residues which form disulfide bonds (three bonds in each EGF domain: C_1_-C_3_, C_2_-C_4_ and C_5_-C_6_) (Figure 1), and together with the calcium-binding region (Figure 1), are critical to the stability of the resulting structure [11,12]. In vitro studies have shown that missense variants in the calcium-binding region (Figure 1) led to increased proteolytic vulnerability of recombinant fibrillin fragments [13,14,15]. It has been also suggested by our group and by other studies [11,12,13,15] that variants in cysteines and amino acids in the calcium-binding region of the cbEGF-like domains disrupt the stability of the domain, and they are predicted to disturb calcium from binding properly since pathogenic variants in these amino acids result in Marfan syndrome phenotype. Sharing structural homology with fibrillins are the latent TGFβ-binding proteins (LTBPs), which are also found in the ECM.

In human tissues, fibrillins coalesce to form microfibrils and typically provide a template structure for the deposition of tropoelastin (the precursor to elastin). These microfibrils play several roles which are tissue-dependent, including providing structural support and elasticity [19].

Fibrillin microfibril formation and deposition is a complex process and occurs in association with other ECM proteins such as the LTBPs, fibronectin and ADAMTS proteases. There are four LTBP isoforms, three of which (LTBP-1, -3 and -4) bind fibrillin and have been demonstrated to facilitate TGFβ sequestration by fibrillin microfibrils [20,21,22].

Fibrillin-1 is the major component of the human zonule [1,23]. Additional elements abundant in the human zonule include LTBP-2, ADAMTSL6, microfibrillar-associated protein 2 (MFAP-2) and EMILIN-1 [1]. LTBP-2, the only LTBP isoform which does not bind TGFβ, co-localises with fibrrilin-1 and is important to zonular structure and integrity.

### Zonular Structure and Physiology

The zonular fibres originate at the pars plana and extend anteriorly to the ciliary body. They then attach to the ciliary body in the grooves of the ciliary processes, from which they extend inwards towards the lens capsule. There are several attachments of the zonule: the anterior equatorial lens capsule, the posterior equatorial lens capsule and an attachment at Wieger’s ligament (between the posterior pole of the lens and the anterior hyaloid) known as the vitreous zonule. Thus, the zonular fibres can be grouped into the anterior, posterior and the vitreous portions.

Investigation of the substructure of the bovine zonular fibres has revealed an organisation which resembles that of muscle fibres [24]. Thousands of individual fibrillin microfibrils are grouped together in fascicles, which in turn coalesce to form larger zonular fibres. 

The adult human ciliary zonule is composed primarily of fibrillin-1 microfibrils, and although free of elastin, it exhibits a significant degree of elasticity with an elastic modulus similar to that of elastin-containing tissues [25,26,27]. There exist crosslinking elements between parallel fibrillin microfibrils [24,28], potentially composed of LTBP-2 [29] or ADAMTSL4 [30], which may serve to pack individual microfibrils together and provide zonular stability. Fibrillin-2 and fibrillin-3 are also present in the developing zonule but are largely absent postnatally in humans [31].

## 3. Clinical Manifestations and Diagnostic Approach

Patients presenting with zonulopathies need to be stratified on clinical grounds initially. Zonulopathy may take a range of clinical manifestations, ranging from a subclinical phenotype to frank EL—complete dislocation of the lens. Trauma is the most common cause of EL. Absence of or mild trauma resulting in EL should raise suspicion of an underlying genetic condition.

### 3.1. Subclinical Zonulopathy

Mild or subclinical zonulopathy is typically not apparent on slit-lamp biomicroscopy but may become apparent during cataract surgery or post-operatively as late intraocular lens dislocation. Intraoperative signs of zonular weakness include floppy iris, posterior capsular laxity and zonular dehiscence, all of which make the procedure more difficult. Typically, in the absence of trauma, subclinical zonulopathy is secondary to pseudoexfoliation syndrome (PXF) [32].

Pre-operatively, the presence of exfoliative material (XFM) in the anterior segment will alert the clinician to PXF, but this is itself often undetectable clinically. Histopathological data suggest that 51.2% of patients may not be diagnosed clinically [33] and fellow “uninvolved” eyes exhibit XFM in patients with clinically unilateral PXF [34]. Additionally, the presence of XFM does not itself correlate with the degree of zonular damage, with bilateral damage demonstrated on ultrasound biomicroscopy in patients with clinically unilateral PXF [35]. As mild zonulopathy is often made manifest during cataract surgery, this cohort of patients is typically older and genetic testing for syndromic disease is not necessarily warranted.

### 3.2. Zonulopathy Manifest as Ectopia Lentis

EL represents the clinical manifestation of severe zonulopathy. Additionally, EL encompasses a range of phenotypes from subluxation to frank dislocation into the vitreous cavity or anterior chamber. Whilst the aetiology of spontaneous EL is broad (Table 1), the predominant cause is an autosomal dominant variant in *FBN1* in 87.3% of cases, most commonly manifested as Marfan syndrome (MFS) (OMIM # 154700) [3,36].

Non-ocular clinical features include increased limb and digit length, highly arched palate and aortic root dilatation which may result in life-threatening dissection. The prevalence of MFS ranges between 6-10 and 100,000 with an increasing incidence [37]. In the eye, MFS is associated with high myopia and EL. Prevalence of EL amongst MFS patients is estimated to be between 30.2 and 62.1% [33,34]. As a result of this, over 50% of patients with MFS are first highlighted by ophthalmologists [16].

Diagnosis of MFS is based on the revised Ghent criteria [2,35]. To summarise, MFS can be diagnosed clinically if the following conditions apply:

(1) In the presence of confirmed MFS family history, the patient exhibits either EL, aortic root dilatation or a systemic score > 7.
(2) In the absence of confirmed MFS family history, the patient exhibits aortic root dilatation in combination with either EL, a systemic score >7 or a known causative *FBN1* variant.
(3) In the absence of confirmed MFS family history, the patient exhibits EL and a known causative *FBN1* variant.

Thus, EL is a clinically important feature of MFS, is often pivotal for its diagnosis and must be necessarily suspected in spontaneous EL. Other connective tissue disorders, such as Ehlers–Danlos (EDS) and Loeys–Dietz syndrome (LDS), may present with similar phenotypes to MFS. LDS, in particular, is phenotypically similar to MFS and EL is considered a discriminating feature [38].

The next most important genetic cause is a variant to *ADAMTSL4* in 6.9% of cases [3] manifesting as isolated EL without extraocular involvement. Therefore, in cases of EL not satisfying the Ghent criteria and without extraocular features, an *ADAMTSL4* variant should be suspected. It is important to note that given the Ghent criteria, cases of isolated EL may be diagnosed as MFS if a previously described *FBN1* mutation is identified [35].

Given the broad range of genes implicated in EL, in addition to the overlapping phenotypes, genetic screening for genes known to cause EL (Table 1) should be necessarily performed. Due to the possibility of a syndromic disorder with potentially life-threatening consequences, systemic investigation is warranted.

The pathophysiology of genetic disorders underlying zonulopathy may be thought of as ECM disorders due to (1) pathogenic variants in *FBN1*, which is the major component of the zonule, (2) genetic variants affecting zonular integrity and attachment, and (3) genetic variants resulting in progressive zonular weakness through indirect action on the ECM components of the zonule. As such severity of the lens phenotype (i.e., EL or subclinical), in combination with additional ophthalmic and systemic features, age of onset and family history may suggest the likely underlying aetiology.

## 4. Ectopia Lentis and *FBN1*

As already stated, the predominant cause of EL is an autosomal dominant variant in *FBN1,* most commonly manifested as MFS (OMIM # 154700) [3,38].

The genotype–phenotype correlation of *FBN1* variants in MFS has been investigated [39,40,41,42]. These have suggested that in-frame variants affecting the cysteine residue of *FBN1* are more likely to lead to EL when compared to premature stop codons or in-frame variants which do not affect the *FBN1* cysteine content. Additionally, in patients exhibiting EL variants resulting in a cysteine change (+ or −), the degree of EL may be more severe. The cysteine residues found in cb-EGF domains of *FBN1* form disulfide bonds [16] and are critical in determining the resultant folding of the protein.

Additionally, neonatal MFS—a life-threatening form of MFS manifesting with EL and a severe cardiac phenotype—has been associated with variants located in exons 24-40 [42], and variants resulting in PTC carry greater risk for aortic dilatation and other cardiovascular features [37]. Thus, establishing genotype–phenotype relationships in MFS may allow for the stratification of patients at high risk of developing life-threatening pathology.

## 5. Disorders Affecting Zonular Integrity and Attachment

### 5.1. The ADAMTS Superfamily

**Figure 2:**
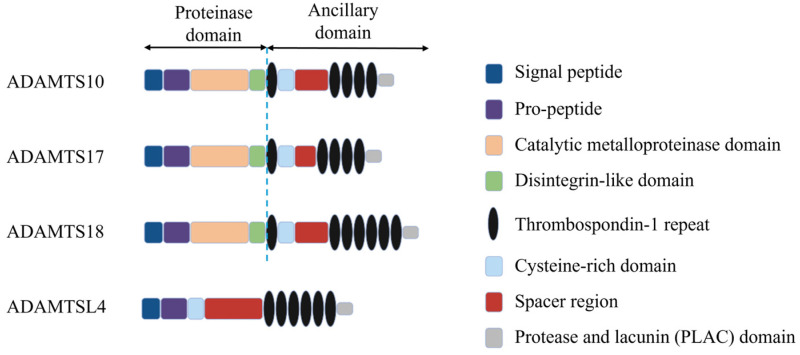
Structure of ADAMTS superfamily proteins implicated in ectopia lentis. ADAMTS proteins share a structurally similar N-terminal proteinase domain and differ in their C-terminal ancillary domain. ADAMTS-like (ADAMTSL) proteins are structurally similar to ADAMTS proteins but lack the catalytic domains.

In addition to MFS, causes of spontaneous EL may be systemic syndromes (e.g., Weill–Marchesani syndrome), or isolated ophthalmic disorders (e.g., isolated ectopia lentis) but are all typically ECM disorders directly affecting the components of the zonule itself or its development. In the case of an autosomal recessive mode of inheritance, the underlying genetic cause is often due to a variant to one of several ADAMTS (A Disintegrin-like And Metalloprotease with ThromboSpondin type 1 motif) genes, most commonly *ADAMTSL4*, but also *ADAMTS10*, *ADAMTS17* and *ADAMTS18*. The general structure of ADAMTS proteases can be seen in Figure 2, with each protein exhibiting a structurally similar proteinase domain and a variable ancillary domain. ADAMTSL proteins are structurally similar to ADAMTS proteases, but they lack their catalytic activity [43].

Given that the aforementioned *ADAMTS* genes and *FBN1* have all been implicated in conditions causing EL, this suggests that they are closely involved in the development and maintenance of the zonule. Table 2 contains a summary of the *ADAMTS* gene variants involved in EL.

### 5.2. ADAMTSL4

*ADAMTSL4* variants are manifested as isolated EL [47,48,49,50,53,54], in which EL occurs without extraocular involvement. Previous case series suggest that patients presenting with EL due to variants in *ADAMTSL4* are diagnosed at an early age—often before the age of 4 [61,63]. Conversely, the literature suggests that MFS is often diagnosed later at around the age of 20 [3,37], though EL in these patients may also be diagnosed early [3]. This suggests that *ADAMTSL4* is fundamental to zonular development.

*ADAMTSL4* is widely expressed throughout the eye, including the ciliary body stroma and the equatorial lens periphery [30,64,65]. The presence of ADAMTSL4 also enhances fibrillin microfibril deposition in fibroblasts [64]. There has been shown to be detachment of the zonule from the lens capsule in juvenile ADAMTSL4-deficient mice [30]. This suggests that ADAMTSL4 may be important for anchoring the zonules to the lens capsule and perhaps explaining the isolated and more severe form of EL with variants in *ADAMTSL4* [61]. Variants in *ADAMTLS4* have also been implicated in extraocular disruption of connective tissue of the cranial sutures, causing craniosynostotis [66].

The preponderance of variants in *ADAMTSL4* causing isolated EL have been nonsense variants resulting in truncated protein [59,60,61,62,63]. Several variants have been hypothesised to abolish the thrombospondin-1 repeat domains resulting in impaired anchoring to the ECM [61,62], consistent with *ADAMTSL4* playing a role in anchoring the zonule to the lens.

### 5.3. ADAMTS10 and ADAMTS17

Variants in several ADAMTS genes have been shown to lead to acromelic dysplasias, a group of connective tissue disorders which share the characteristic features of short stature and abnormalities of the hands and feet [67]. Of particular importance to the eye, recessive variants in ADAMTS10 [39,40,41] and ADAMTS17 [41,42] may lead to Weill–Marchesani syndrome (WMS), which exhibits EL and microspherophakia as features in addition to musculoskeletal and cardiovascular abnormalities.

The relationship of ADAMTS10 and ADAMTS17 with fibrillin microfibrils has been investigated. ADAMTS10 may be necessary for fibrillin-1 microfibril formation with both ADAMTS10 and ADAMTS17 appearing to regulate fibrillin-2 inclusion in microfibrils in the adult zonule—which in humans is formed primarily of fibrillin-1 and is deficient in fibrillin-2. ADAMTS10 binds and localises to fibrillin-1 [67] and accelerates fibrillin-1 microfibril formation [67]. In vivo, it does not undergo furin processing and has minimal activity as a fibrillin-1 protease, but has been shown to exhibit increased activity in cleaving fibrillin-1 with restoration of the consensus furin-processing site [67]. Additionally, mouse models have shown *ADAMTS10* to be widely expressed in ocular tissues during embryonic development including the non-pigmented ciliary epithelium and the lens epithelium [39]. ADAMTS17, also present in the eye in both humans [40] and mice [41], undergoes autocatalysis and has been shown to bind to but not cleave fibrillin-1 microfibrils [41].

Variants in *ADAMTS10* causing WMS have predominantly been missense variants resulting in the reduced secretion of the protein product [40,45], with nonsense variants also reported [44]. Affected domains have included the catalytic domain, the signal peptide and the pro-peptide with predicted nonsense-mediated decay, truncated protein product or reduced protein secretion [40,44,45]. Variants in *ADAMTS17* have also predominantly been missense variants, resulting in the predicted absence or truncation of the protein product [40,47,48,49,50,53,54]. Affected domains have included the thrombospondin-1 repeats [40,52], catalytic domain [40,51,53] and the reprolysin domain [49].

Therefore, it appears that variants in both *ADAMTS10* and *ADAMTS17* resulting in WMS are due to loss of either protein, and that both proteins are necessary for zonular integrity. Karoulias et al. have hypothesised that ADAMTS17 may play a role in activating the proteolytic activity of ADAMTS10 following fibrillin-1 binding given that ADAMTS10 is not activated by furin processing [42]. However, exactly how ADAMTS10 and ADAMTS17 interact in vivo is yet to be elucidated.

### 5.4. ADAMTS18

*ADAMTS18* has been implicated in cases of microcornea myopic chorioretinal atrophy and telecanthus (MMCAT) and Knobloch syndrome exhibiting EL [57,58].

Variants in *ADAMTS18* leading to EL consist of nonsense variants resulting in an almost complete loss of protein [55,56,58] or missense variants in highly conserved regions of the disintegrin [55,56], pro- [57] and cysteine-rich domains [58]. This suggests that, for *ADAMTS18* variants to cause EL, there must be loss of protein function. However, how *ADAMTS18* contributes to zonular integrity is unclear.

**Figure 3:**
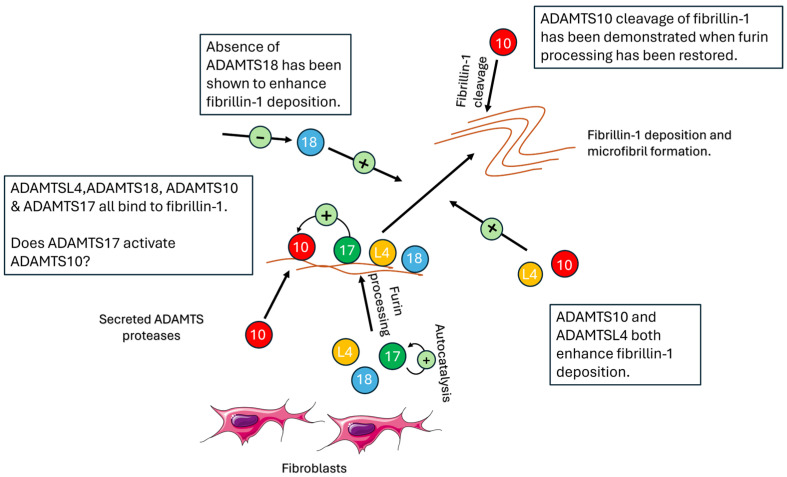
Summary of the interactions between ADAMTS proteins and fibrillin-1.

Mouse models have demonstrated *ADAMTS18* expression in the lens capsule [57,68]. Additionally, breaks in the posterior lens capsule with extrusion of lens contents have been observed in *Adamts18*^−/−^ mice [68]. Extraocularly, *Adamts18*^−/−^ mice have been shown to exhibit fewer and shorter bronchi with associated increased levels of fibrillin-1 and fibrillin-2 deposition [69]. Whilst fibrillin-1 has been shown to be a substrate of ADAMTS18 in the lungs [69], its substrate in the eye has yet to be elucidated, although it is possible that it plays a role in the zonular attachments in the lens capsule through regulation of fibrillin-1 deposition. Alternatively, the mechanism for EL in ADAMTS18 deficiency may result from the disruption of the lens capsule apart from actions of ADAMTS18 on fibrillin-1 as a substrate (Figure 3).

### 5.5. Knobloch Syndrome

Knobloch syndrome is a disease of autosomal recessive inheritance and is characterised predominantly by ocular abnormalities such as high myopia, retinal degeneration and lens subluxation. It is principally caused by variants in *COL18A1* found on chromosome 21q22.3 (OMIM *120328) [70].

*COL18A1* has two promoters which produce three different transcripts of collagen XVII, each with varying lengths. Collagen XVIII is a component of many basement membranes and is also found in the lens capsule and ciliary body [71,72], both insertion sites of the zonule. Collagen XVIII has been shown to have a polar orientation, with the C-terminal portion expressed within the lamina densa and the N-terminal non-collagenous domain of collagen XVIII (NC11) expressed in the sub lamina densa [73].

*COL18A1* knockout mice have been shown to exhibit separation of the vitreous from the internal limiting membrane, suggesting that collagen XVIII may potentially play an anchoring role at the vitreoretinal interface as well [72]. Given that EL is a feature of Knobloch syndrome, it may be that certain *COL18A1* variants result in the disruption of zonular anchoring to the lens capsule or ciliary body due to disrupted interactions between collagen XVIII and fibrillin-1 in the zonule. However, there has been no demonstration of any interaction between collagen XVIII and fibrillin-1.

A single case of a 3-year-old girl with a variant in *VSX2* with a phenotype including EL and resembling Knobloch syndrome has been reported [74]. Further investigations into the role of VSX2, the ECM and the zonules would be warranted.

## 6. Disorders Indirectly Affecting Zonular Constituents

### 6.1. Homocystinuria

Homocystinuria is an inherited disorder of metabolism in which levels of the amino acid homocysteine are raised. Defects to the cystathionine β-synthase enzyme, responsible for the conversion of homocysteine to cystathionine in the transulfuration pathway, are the most common cause of homocystinuria [75]. Diagnosis is often made through newborn screening programmes. Previous case series have suggested that patients diagnosed and treated soon after birth may not experience EL, compared to late-diagnosed patients [76,77]. Clinically, the age at which EL occurs is related to treatment, with treatment-naive or poorly responsive patients exhibiting EL earlier than treatment-responsive patients [78,79,80].

As homocystinuria shares many features with Marfan syndrome, it has been hypothesised that raised homocysteine levels resulted in disruption to fibrillin-1 microfibrils [81,82].

Elevated homocysteine levels have been shown to have various effects on fibrillin-1. In homocystinuria, there is a concentration-dependent reduction in fibrillin-1 cb-EGF domain disulfide bonds, resulting in the alteration of the secondary structure of fibrillin1 and increased susceptibility to proteolysis [81,82]. The C- and N-terminal self-interaction, an important step for fibrillin microfibril formation, is impaired [83]. Human skin fibroblasts cultured in elevated homocysteine deposit fewer fibrillin-1 microfibrils [83]. Thus, elevated homocysteine has consequences for the ciliary zonule via its effect on fibrillin-1 structure and function.

### 6.2. ASPH

Traboulsi syndrome (OMIM: 601552) is a rare cause of spontaneous EL caused by variants to the gene *ASPH* [84], and has been suggested to be responsible for 0.5% of cases [85]. Along with EL, other features include abnormalities of the anterior segment and facial dysmorphism. *ASPH* encodes the protein asparaginyl β-hydroxylase, which has been shown to have fibrillin-1 and LTBP-2 as substrates [86], and is expressed in the developing mouse lens [84] and in the human non-pigmented ciliary epithelium [85]. *ASPH* variants are hypothesised to modify disulfide bond formation in fibrillin-1 through action on cb-EGF domains [85].

## 7. Pseudoexfoliation

PXF is an age-related disorder of extracellular matrix synthesis and deposition. Prevalence is known to vary in different populations [32,87]. Whilst a systemic disease, it mainly manifests itself in the eyes and is a common cause of both glaucoma [88] and zonular weakness [5,32,89].

In PXF, there is a production of exfoliative material (XFM) by non-pigmented ciliary epithelial cells, lens epithelial cells and various other cell types in the anterior segment [90]. XFM deposits have been shown to consist of various ECM-related proteins including fibrillin-1 [91,92], LTBP2 [92,93], lysyl oxidase-like1 (LOXL1) [93,94], apolipoprotein E [93] and Clusterin [91,93], among others. PXF does not cause zonulopathy directly through alterations in zonular formation or structure. Rather, as production of XFM increases with age, deposition onto the zonule results in progressive weakening which may manifest clinically as phacodonesis. PXF patients are therefore at greater risk of complications during cataract surgery and post-operative IOL dislocation [5,32].

The genetic associations of PXF have been investigated. Genome-wide association studies have suggested variants in *LOXL1* as the most significant risk factor of PXF [76,95]. In particular, two single nucleotide polymorphisms in exon 1 were found to confer increased risk in Scandinavian populations [95], and this association was replicated in other Caucasian populations [77,78], and also, in an American study [79] where patients from America (mostly of European background) and from 12 European countries were studied. However, the risk allele was reversed in certain ethnic groups, including Japanese [76,80,96] and Black South Africans [97]. Therefore, the genesis of PXF is likely multifactorial with genetic and environmental [83,98] factors contributing.

LOXL1 plays an important role in the formation of elastic fibres [84], being responsible for the maturation of tropoelastin into elastin. LOXL1 is also present in the zonule, making up 0.25% of the proteome, where it has been suggested to act as a crosslinker [1,85]. LOXL1 levels have been shown to be increased in fibrotic disorders and excessive ECM deposition of the liver [86], heart [99] and other organs. In early PXF, expression of *LOXL1* is increased in human eyes and is associated with XFM production, whereas expression is decreased in later stages [94]. This suggests that abnormally elevated levels of *LOXL1* expression may be involved in driving production of XFM, with subsequent down-regulation to attenuate this process. Experimentally, transgenic mice with increased lens epithelial *Loxl1* expression showed no difference in zonular structure compared to wild-type controls and did not produce XFM, though they did exhibit production of LOXL1 aggregates on the lens [85]. A zebrafish model of PXF has compared *loxl*^−/−^, *loxl*^+/−^ and *loxl*^+/+^ genotypes [100]. The authors found *loxl*^−/−^ and *loxl*^+/−^ to have morphological defects in the zonule and accumulation of pearly white particles with age. However, these particles could not be confirmed to be similar to XFM compositionally.

There is evidence that autophagy is impaired in PXF cells, potentially leading to an accumulation of misfolded proteins, in particular LOXL1 [101]. Additionally, studies of differential gene expression in PXF eyes versus healthy controls have demonstrated overexpression of elastic microfibrillar components [102]. Conversely, there is down-regulation of stress-related cytoprotective proteins, including the chaperone Clusterin, ubiquitin-conjugating enzymes and glutathione-S-transferases [103]. Thus, there is an increase in LOXL1 misfolding, with impairment of the molecular chaperone Clusterin to prevent aggregation of misfolded proteins, resulting in a build-up of XFM.

In summary, the pathophysiology of pseudoexfoliation is multifactorial, with *LOXL1* variants being the most important genetic risk factor. There is increased expression of *LOXL1* early in the disease process, associated with LOXL1 misfolding, impaired autophagy and production of XFM, resulting in deposition on the zonules and progressive zonular weakness.

## 8. Discussion

In this review, we presented the framework of zonulopathies as essentially disorders of the ECM for the understanding of the underlying pathogenetic mechanisms of EL. Using this framework, genetic disorders resulting in EL can be understood to arise due to (1) pathogenic variants in fibrillin-1, which is the major component of the zonule, (2) genetic variants affecting zonular integrity and attachment and (3) genetic variants resulting in progressive zonular weakness through indirect action on the ECM components of the zonule.

Whilst this framework is not entirely novel, as the variants discussed in this review are well recognised to have implications for ECM function, zonulopathies are not usually referred to as essentially ECM disorders. This may be for the following reasons: (1) EL may be the first sign of a systemic condition, but may also present without systemic involvement as isolated EL, and (2) zonular weakness may result from surgical intervention or trauma and the zonules may weaken with age [27].

Previous reviews have classified genetic causes of EL into MFS, autosomal recessive EL and autosomal recessive isolated EL [23] and into those with and without systemic manifestations [104]. However, the strength of this framework is that it recognises the relationship played between fibrillin-1 and the protein products of the implicated genes. Furthermore, the zonule has long been recognised as being primarily composed of ECM proteins [1]. As such, this framework provides insight into the underlying pathogenetic mechanisms of inherited zonulopathies and the structure of the healthy zonule.

Much of the emphasis in the literature has been to characterise the genotype–phenotype relationship of the genetic variants implicated in EL, especially in *FBN1* [3,61,105]. With regards to EL, it is understandable that genetic variants affecting fibrillin-1 or proteins intimately involved in zonular structure and integrity, will be more likely to result in spontaneous EL whereas those causing more indirect effects to the zonule will result in progressive zonular weakness.

**Figure 4:**
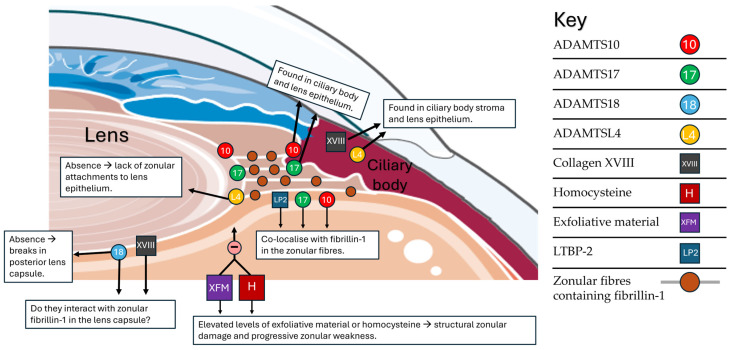
Summary of important proteins implicated in the ectopia lentis phenotype. Picture representing part of the lens on the left and part of the ciliary body on the right. The lines between the lens and ciliary body represent the zonular fibres. Note that the zonular fibres are composed primarily of fibrillin-1. The protein names are placed either in a circle (with number) or a square (names): LTBP-2 = latent TGFβ-binding protein 2; XVIII = collagen XVIII; 10 = ADAMTS10; 17 = ADAMTS17; 18 = ADAMTS18; L4 = ADAMTSL4; XFM = pseudoexfoliative material.

A weakness of this framework is that it does not account for the Anterior Segment Dysgeneses (ASDGs) as a cause for EL. ASDGs are a group of congenital heterogeneous disorders affecting the structures of the anterior segment, including the trabecular meshwork, cornea, iris, lens and ciliary body. Features of ASGDs include aniridia, buphthalmos secondary to raised intraocular pressure, anterior synechiae, cataract and EL, though EL is not a defining feature. Unlike the other causes of EL mentioned in this review, ASGDs are disorders of development and not of the ECM. As such, implicated genes causing ASGD and EL, such as *PAX6* [106,107] and *CPAMD8* [108], are not typically associated with extraocular connective tissue abnormalities. However, the purpose of this framework is not to be a completely comprehensive classification of the genetic causes of EL, but to provide insight into zonulopathies in which EL and/or progressive zonular weakness is a defining or characteristic feature(Figure 4).

## 9. Future Directions

A wide range of opportunities for research and gene discovery for EL remains available. Improved technologies like short/long reads next generation sequencing (NGS) (especially Whole Genome Sequencing [WGS]) will make it possible to cover regions that, before, were hardly studied in EL, and gene editing (Prime Editing) will help to manipulate the DNA sequence to try to rectify the cause of the EL phenotype.

The usage of NGS technologies has accelerated causative variant detection and discovery of novel genes. There are many EL studies where variants are discovered using second (short reads) generation sequencing [109] but third (long reads) generation sequencing studies need to catch up. The focus on current EL studies remains to detect point variants or short indels, but they often forget to address and study deep intronic regions, intergenic regions and structural variants (including Copy Number Variations [CNVs]). Now that WGS long reads are more accessible with the recent introduction of the PromethION 2 Solo (P2 Solo) sequencer in 2022, the query for variants in deep intronic regions and the evaluation of structural variants like CNVs will be much more standard.

Another technology advancement that EL should take advantage of is the improvement of gene editing with the introduction of “Prime Editing”. Even though CRISPR-Cas9 is very useful, Prime Editing is more precise when it comes to modifications of the DNA sequence, but as well as CRISPR-Cas9, the technology must be improved to reduce off-target effects. This technology can potentially clarify the ambiguity of variants of unknown significance (VUS) where their current significance is not well understood. Animal model studies or cell studies can help improve the variant classification towards a much robust position: pathogenic or benign.

## 10. Conclusions

Genetic disorders resulting in zonulopathy and EL are numerous and are often syndromic systemic disorders of the ECM. Pathophysiologically, genetic causes of EL share the final common pathway of disrupting fibrillin-1, leading to zonular disruption and EL. In the absence of trauma, EL should raise a high suspicion for MFS and, as such, it is necessary for the clinician to conduct a targeted systems review and family history, in addition to an ophthalmic examination. Given the broad aetiology of EL, screening variants in known causative genes must be included in investigations.

Further work to identify variants and elucidate the pathophysiology of how these affect the zonule is of particular importance to the Ophthalmologist, the Visual Scientist and those interested in the ECM.

## Figures and Tables

**Table 1:** Genes associated with ectopia lentis.

Disease/Syndrome	Mode of Inheritance	Gene
Hyperlysinaemia type 1	AR	*AASS*
Craniosynostosis with ectopia lentis	AR	*ADAMTSL4*
Ectopia lentis et pupillae	AR	*ADAMTSL4*
Ectopia lentis type 2	AR	*ADAMTSL4*
Weill–Marchesani syndrome type 1	AR	*ADAMTS10*
Weill–Marchesani syndrome type 4	AR	*ADAMTS17*
Knobloch syndrome	AR	*ADAMTS18*
Microcornea, myopic chorioretinal atrophy and telecanthus	AR	*ADAMTS18*
Traboulsi syndrome	AR	*ASPH*
Microphthalmia, syndromic 2	XLD	*BCOR*
Homocystinuria due to cystathionine β-synthase deficiency	AR	*CBS*
Classic Ehlers–Danlos syndrome type 1	AD	*COL5A1*
Classic Ehlers–Danlos syndrome type 2	AD	*COL5A2*
Knobloch syndrome	AR	*COL18A1*
Anterior segment dysgenesis 8	AR	*CPAMD8*
Ectopia lentis type 1	AD	*FBN1*
Marfan lipodystrophy syndrome	AD	*FBN1*
Weill–Marchesani syndrome type 2	AD	*FBN1*
Exfoliation syndrome	AD	*LOXL1*
Glaucoma 3, Primary Congenital, D	AR	*LTBP2*
Microspherophakia and/or megalocornea, with ectopia lentis and with or without secondary glaucoma	AR	*LTBP2*
Weill–Marchesani syndrome type 3	AR	*LTBP2*
High myopia with cataract and vitreoretinal degeneration	AR	*P3H2*
Anterior segment dysgenesis 5	AD	*PAX6*
Anterior segment dysgenesis 8	AR	*CPAMD8*
Focal dermal hypoplasia	XLD	*PORCN*
Isolated sulphite oxidase deficiency	AR	*SUOX*
Cohen syndrome	AR	*VPS13B*
High myopia, EL, cone-rod dystrophy	AR	*VSX2*
Knobloch syndrome	AR	*VSX2*

The first column in the table shows the name of the disease or syndrome that is associated with a gene in the third column. Note that some genes are associated with several diseases and/or syndromes. The second column shows the mode of inheritance: AD (autosomal dominant); AR (autosomal recessive); XLD (X-linked).

**Table 2:** Variants in ADAMTS genes resulting in ectopia lentis.

Gene	Phenotype	Variants
*ADAMTS10*	WMS	c.709C>T; p.R237* [44]
	WMS	c.1190+1G>A [44]
	WMS	c.1553G>A; p.G518D [40]
	WMS	c.2098G>T; p.G700C [40]
	WMS	c.73G>A; p.A25T [45]
	WMS	c.952C>T; p.Q318* [45]
	WMS	c.41T>A; p.L14Q [46]
*ADAMTS17*	WMS	c.625delG; p.D218Tfs*41 [47,48]
	WMS	c.873+1G>T [49]
	WMS	c.2458_2459insG; p.E820Gfs*23 [40]
	WMS	c.760C>T; p.Q254* [40]
	WMS	c.1721+1G>A [40]
	WMS	c.1297C>T, p.R433* [50]
	WMS	c.2948C>T, p.T983M [50]
	WMS	c.1322+2T>C [50]
	WMS	c.1716C>G, p.N572K [50]
	WMS	c.1630G>A, p.G544R [50]
	WMS	c.1669C>T, p.R557* [50]
	WMS	c.1027A>G, p.T343A [51]
	WMS	c.3068G>A, p.C1023Y [52]
	WMS	c.1051A>T, p.K351* [53]
	WMS	c.1051_1053delAAGinsTAA, p.K351* [54]
*ADAMTS18*	MMCAT	c.1731C>G; p.C577W [55,56]
	MMCAT	c.2065G>T; p.E689* [55,56]
	MMCAT	c.606T>C; p.L202P [55,56]
	MMCAT	c.97C>T; p.Q33* [55,56]
	KS	c.536C>T; p.S179L [57]
	MMCAT	c.1067T>A; p.L356* [58]
	MMCAT	c.2159G>C; p.C720S [58]
	MMCAT	c.1952G>A; p.R651Q [58]
*ADAMTSL4*	IEL	c.1785T>G; p.Y595* [59]
	IEL	c.79-1G>A [60]
	IEL	c.767_786del; p.Q256Pfs*38 [61,62]
	IEL	c.2008C>T; p.R670* [62]
	IEL	c.1960C>T; p.P654S [62]
	IEL	c.826_836del; p.R276Sfs*21 [62]
	IEL	c.936G>A; p.R309Q [62]
	IEL	c.2177+4A>G [54]
	IEL	c.3161A>G; p.Y1054C [62]
	IEL	c.3153C>A; p.Y1051* [62]
	IEL	c.759_778del20; p.Q256Pfs*38 [63]
	ELP	c.293delG; p.G99Afs*34 [61]
	ELP	c.925C>T; p.R309* [61]
	ELP	c.237delC; p.P80Rfs*53 [61]
	ELP	c.767_786del20; p.Q256P*38 [61]

IEL = Isolated ectopia lentis, ELP = Ectopia lentis et pupillae, WMS = Weill–Marchesani syndrome, MMCAT = Microcornia, myopic chorioretinal atrophy and telecanthus, KS = Knobloch syndrome.
